# The Behavioral Level of Emotional Intelligence and Its Measurement

**DOI:** 10.3389/fpsyg.2018.01438

**Published:** 2018-08-13

**Authors:** Richard E. Boyatzis

**Affiliations:** Department of Organizational Behavior, Case Western Reserve University, Cleveland, OH, United States

**Keywords:** emotional intelligence, competencies, multisource assessment, social intelligence, behavioral assessment

## Abstract

Emotional Intelligence (EI) is now widely used in organizations and graduate schools with an increase in published research supporting it. Discussion about EI whether based on measures or theory has given little distinction as to behavioral EI (i.e., how does EI appear in a person’s actions). This results in spurious conflicts about the validity of the different theories or measures which likely limit predicting managerial and leadership effectiveness, engagement, innovation and organizational citizenship. By adding a behavioral level, the concept of EI could relate to work and life outcomes beyond general mental ability and personality traits, avoid some of the criticisms while providing a more holistic theory of EI. As such, EI exists within personality as a performance trait or ability, *and* a self-schema self-image and trait, *and* a set of behaviors (i.e., competencies). The main contribution of this establishing the behavioral EI with a multi-level theory, while explaining how to assess it, the benefits of such a concept and its psychometric validity and challenges. The history and assortment of validation studies will illustrate that measures can rigorously and effectively assess the behavioral level of EI.

## Introduction

Emotional Intelligence (EI) is now widely used in organizations and graduate schools with an increase in published research supporting it. Discussion about EI whether based on measures or theory has given little distinction as to behavioral EI (i.e., how does EI appear in a person’s actions). The emergent conflicts about the validity of the different theories or measures of EI are a likely limit to predicting managerial and leadership effectiveness, engagement, innovation and organizational citizenship. By adding a behavioral level, the concept of EI could relate to work and life outcomes beyond general mental ability and personality traits, avoid some of the criticisms while providing a more holistic theory of EI. As such, EI exists within personality as a performance trait or ability *and* a self-schema self-image and trait *and* a set of behaviors (i.e., competencies). This paper will focus on the behavioral level of EI. Specifically, the contributions of this paper are: (1) articulating that there is a behavioral level within the structure of EI; (2) review the history and an assortment of validation studies of behavioral EI that illustrate that measures can effectively assess the behavioral level of EI; and (3) highlighting the behavioral level within a multi-level theory of EI. The paper will first review the development of behavioral measures of EI and then explore the distinctive benefits of using a behavioral level measure. This will be followed by proposing a multi-level theory of EI and discussing how it is a domain specific application of a more general multi-level theory of personality. The paper will conclude with a discussion of the measurement challenges at the behavioral level.

## Development of Behavioral Measures of EI

The controversial commentary on EI has often focused on the appropriateness or validity of various measures (Matthews et al., 2002; Boyatzis, 2009). At the center of these disagreements may be the criteria considered relevant for defining EI. Mayer et al. (1999, pp. 269–270) claimed that a concept of EI “(1) should reflect a mental performance rather than preferred ways of behaving; (2) tests of it should show positive correlation with other forms of intelligence; and (3) the measures should increase with experience and age.” Offering an alternative, Boyatzis and Sala (2004) claimed that a concept of EI should be: “(1) behaviorally observable; (2) related to … [specific] neural-endocrine systems; (3) related to life and job outcomes; (4) sufficiently different from other personality constructs … and (5) the measures of the concept … should satisfy the basic criteria for a sound measure.” (p. 148). The need for a link to outcomes also comes from the American Psychological Association’s Task Force on Intelligence (Public Affairs Office, 1997; American Psychological Association [APA], 2018) which claimed that predicting real life outcomes is an important part of the standard against which we should judge an intelligence.

The definition of behavioral EI was defined as “*(a) an emotional, intelligence competency is an ability to recognize, understand, and use emotional information about oneself that leads to or causes effective or superior performance; and (b) a social intelligence competency is the ability to recognize, understand and use emotional information about others that leads to or causes effective or superior performance.”* (Boyatzis, 2009, p. 757) The historical development of the EI competencies helps to explain why the behavioral level of EI is closely tied to work and life outcomes.

### Content Validity

The behavioral approach to EI emerged from two research streams: (1) inductive analysis of criterion-referenced, critical incident interviews against performance; and (2) assessment center coding of simulations. The inductive analysis would begin with identifying outstanding or exceptionally effective people in a specific job and those who were “average” or typical. Using these criterion for sample identification is called an extreme case research design (Boyatzis, 1998). The basis for the criterion classification for management and leadership roles were typically nominations which were seen as more rigorous than ratings or even rankings (Lewin and Zwany, 1976). The nominations were collected from bosses, peers and subordinates to obtain a comprehensive view of the person (Boyatzis, 1982). Occasionally, other data could be used like climate surveys of subordinates, waste reduction for plant managers, sales of retail outlets, and such as the basis for sampling. The outstanding group of people were those that appeared in multiple lists from each of the sources. The average or typical performer group was randomly selected from all of those with no nominations from any source which was always a much larger segment of the population than the outstanding performers. Because of this inductive approach, the cultural and language bias that may be found in questionnaires that do not establish true item and method equivalence across cultures (de Vijver and Tanzer, 2004) was minimized as shown by Sharma (2012) assessing 400 middle level Indian managers.

The critical incident interview, also called the behavioral event interview or behavioral interviewing was an attempt to reconstruct what occurred in specific work situations (Flanagan, 1954; Boyatzis, 1982; Spencer and Spencer, 1993). In this interview protocol, a person was asked, “Tell me about a time you felt effective as a [title of the job being examined].” After recording a 15 min elaboration of the incident, the interview went on to another incident. The elaboration for each incident was developed by asking the person to tell the story, with probes such as, “What happened next? What did you specifically say or do at that moment” What were you thinking or feeling at that time?” The interview would ask for a second incident, often, “Tell me about a time in which you felt ineffective as a [title of the job being examined].” Another effective and ineffective incident were also collected resulting in a total of 4–6 incidents per interview.

Since the behavior shown in the actual situation of the person’s work, the competencies were compiled into a codebook that differentiated outstanding versus average performers were content valid. It was a part of how they acted in performing the job. They were examined in the context in which they appeared. From the context and the groups of similar behaviors, an underlying intent was determined. These functionally related behaviors and the underlying intent became the definition of the competency (Boyatzis, 2009).

Assessment centers often included audiotaped stress interviews or videotaped group simulations (Thornton and Byham, 1982). Although in the early decades using assessment centers, codes were applied to live observation, after the 1980’s, tapes were used for coding of behavior shown.

By the early 1980’s, the desire was to find ways to capture the behavioral competencies that differentiated effective performers without the many hours involved in collecting the interviews (i.e., audiotaped) or simulations (i.e., videotaped) and the coding. Coding had to be done by reliable coders. Each coder tended to spend 2–3 times the running time of audio or videotape. At least two coders had to review each tape and then reconciliation meetings were conducted to determine a consensus coding. The coders were supposed to be statistically checked for their inter-rater reliability annually. This was a highly labor intensive and therefore costly process. It made it prohibitive to amass sufficiently large sample sizes for multivariate analysis.

The behaviors and underlying intent in each competency were converted into questionnaire items. In the case of the ESCI (Boyatzis and Goleman, 2007), the translation was done by the authors of the test. Scales were constructed from the various behaviors. These were reflective items, not formative, because a person might use the competency somewhat differently in a specific situation. The desire was to assess the most typical behaviors.

The “other” or informant responses to the ESCI 360° assessment provides a view of how others see the focal person acting in various situations. In some applications, boss, peers and subordinates are solicited as informants. In other studies, these sources may be supplemented by clients/customers, spouse or partner, friends, and such.

The generation of the items in the behavioral approach is important because it explains why there is not the same pattern of statistical relationship to personality traits found in research using self-assessment (Joseph et al., 2015) or statistical relationship to General Mental Ability (GMA) or cognitive intelligence measures found with ability assessments, like the Mayer Salovey Caruso Emotional Intelligence Test (MSCEIT). The items in the ESCI are the actions shown by a person rather than the typical method for identifying items which begin with a scholar’s theory as to what behavior, feelings or attitudes should reflect the theoretical element to be captured by an item. When items are constructed for self-assessment, there is increased possibility of overlap with other self-perceived aspects of one’s personality, such as their personality traits which is why other approaches to measuring EI may show lower association or prediction of job and life outcomes.

### Emergence of the Competency Codes

As was said above, one of the sources of the behavioral level of EI measurement was coding of audiotapes of critical incidents or videotapes of simulations. The earliest documentation of validity of such coding of competencies against performance appeared in Boyatzis (1982). His results were based on a sample of 6 large companies and 6 large government agencies. Twelve of the sixteen competencies differentiating more effective from less effective managers in this sample were later labeled as EI competencies (Boyatzis, 2009).

A variety of other studies showed validity of behavioral EI against various performance measures. Among 53 entrepreneurs in Italy, Camuffo et al. (2012) showed more effective entrepreneurs as compared to the less effective on 25 behavioral EI competencies versus 8 cognitive ones. For 35 research and development managers at NASA (i.e., section chiefs), Dreyfus (2008) reported that ten out of twelve competencies that distinguished effective performance were behavioral EI. Assessing Chinese and Indian CEOs, Gutierrez et al. (2012) examined 32 Indian and 38 Chinese CEOs and found that all of the 11 competencies identified in the Indian better performers were behavioral EI, and 6 of the 8 competencies identified as distinguishing better performance of the Chinese CEOs were behavioral EI. A more complete analysis and discussion of this study can be found in Spencer et al. (2007). Williams (2008) reported all 20 of the competencies examined were behavioral EI among 20 elementary, middle, and high school principals in an urban school district. Assessing a 47 managers from various companies in Europe, and 15 knowledge works from those same firms, Ryan et al. (2009) reported that 11 of the 13 and 11 of the 12 competencies distinguishing effective performance were behavioral EI, in the two samples respectively.

### A Behavioral Model of EI and SI

The Emotional and Social Competency Inventory (ESCI) was developed to reflect not just the intrapersonal recognition and management of one’s own emotions but also how they influence interpersonal interactions with other people, the recognition and management of others’ emotions. It is the most used behavioral measure of EI in practice and for which the most published studies have occurred. Integrating the codebooks from hundreds of behavioral, inductive studies, items were generated for what appeared to be the most generic competencies (i.e., consistently validated in competency studies across jobs, industries, sectors and countries) in the EI and SI domains. The resulting model included twelve competencies in four clusters representing emotional and social intelligence with two clusters each (Goleman, 1995; Boyatzis and Goleman, 2007; Boyatzis, 2009).

Theoretically determined, EI consists of: (1) the self-awareness cluster concerns knowing one’s internal states, preferences, resources, and intuitions, consisting of emotional self-awareness (i.e., recognizing one’s emotions and their effects); and (2) the self-management cluster refers to managing one’s internal states, impulses, and resources consisting of emotional self-control (i.e., keeping disruptive emotions and impulses in check), adaptability (i.e., flexibility in handling change), achievement orientation (i.e., striving to improve or meeting a standard of excellence), and positive outlook (i.e., seeing the positive aspects of things and the future). Social intelligence consists of: (1) the social awareness cluster refers to how people handle relationships and awareness of others’ feelings, needs, and concerns, consisting of empathy (i.e., sensing others’ feelings and perspectives, and taking an active interest in their concerns), and organizational awareness (i.e., reading a group’s emotional currents and power relationships); and (2) the relationship management cluster concerns the skill or adeptness at inducing desirable responses in others, consisting of coach and mentor (i.e., sensing others’ development needs and bolstering their abilities), inspirational leadership (i.e., inspiring and guiding individuals and groups), influence (i.e., wielding effective tactics for persuasion), conflict management (i.e., negotiating and resolving disagreements), and teamwork (i.e., working with others toward shared goals).

Not all of the competencies appearing in the inductive studies discussed earlier were classified as EI. Competencies having to do with cognitive or analytic processing were not included, such as diagnostic reasoning, conceptual reasoning, synthetic reasoning. Other competencies, such as risk orientation and information collection were also deemed not central to EI by the authors. From assessment centers, a number of the “dimensions” which the authors used to code behavior were also not considered EI, such as decision making. Such dimensions were considered by the author to be job tasks and did not satisfy the core requirements of a competency being a part of a person’s capability.

The question raised at times as to whether behavioral EI competencies are even a part of EI (Cherniss, 2010) is answered in part by the content validity of the competencies included in the behavioral EI model. These 12 competencies in the ESCI are the same or closely related to the elements of EI from other theories, measures and scholars. These twelve competencies have considerable conceptual overlap with measures of EI most often appearing in academic journals and books as cited by the Consortium for Research on Emotional Intelligence. The comparison is shown in **Table 1**. This provides another perspective on the content validity of the behavioral EI twelve scales as aspects of EI and not merely other realms of human behavior.

**Table 1:** Content comparison of behavioral model EI competencies (ESCI) with the scales of other measures.

Behavioral Model ESCI^5^	EQ-i Scales^1^	MSCEIT Branches^6^	WLEIS^2^	TEIQue^3^	GENOS^4^
Emotional self-awareness	Emotional self-awareness	Understanding	Self-emotion appraisal	Emotion perception	Self awareness
Emotional self-control	Impulse control	Managing	Regulation of emotions	Impulsiveness stress management	Self-management
Adaptability	Flexibility	Managing		Adaptability	Self-management
Achievement Orientation	Self-actualization	Managing	Use of emotions	Self-motivation	
Positive outlook	Optimism	Facilitating	Use of emotions	Optimism	
Empathy	Empathy	Understanding	Others’ emotions appraisal	Empathy	Awareness of others
Organizational awareness		Perceiving		Social awareness	
Inspirational Leadership		Managing			Positive influence
Influence	Assertiveness	Managing		Assertiveness	Positive influence
Conflict Management		Managing			
Coach and Mentor		Managing			
Teamwork	Interpersonal	Managing		Relationships	Positive influence

*^1^Bar-On, 1997. ^2^Law et al., 2004; SSEIT Schutte et al., 1998 is not displayed because it yields one EI score. ^3^Petrides and Furnham, 2000. ^4^Gignac, 2008 with edits to skill labels from the GENOS website, 2018. ^5^Boyatzis and Goleman, 2007. ^6^Mayer et al., 2003.*

### Structural Integrity and Validity of a 360° Behavioral Measure

Two versions of the ESCI were developed (Boyatzis, 2009). One was developed for research with working adults, and the other (the ESCI-U) was a university version for use with undergraduates and graduate students. To determine the structural integrity, a sample of 5,761 self-assessments and 62,297 informant assessments of the ESCI and 1,629 self-assessments and 21,288 informant assessments of the ESCI-U were analyzed (Boyatzis et al., 2015b). For each competency for each of the four samples (i.e., self and informant for the ESCI with 6 items in each scale and ESCI-U with 5 items in each scale) internal consistency, reliability, factor structure, and construct validity were all within acceptable parameters. The factor structure was shown in exploratory factor analyses as well as appropriate model fit confirmatory factor analyses: for ESCI self, effective *n* = 4,468, RMSEA = 0.043, CFI = 0.849; for ESCI other, effective *n* = 25,057, RMSEA = 0.044, CFI = 0.910; for ESCI-U self, effective *n* = 1,398, RMSEA = 0.042, CFI = 0.875; for ESCI-U other, effective *n* = 8,981, RMSEA = 0.042, CFI = 0.919. In addition, convergent and discriminant validity was determined using the usual criteria (Boyatzis et al., 2015b) with composite reliabilities of the 12 scales ranging from 0.801 – 0.902 for the ESCI and ESCI-U self and 0.865–0.939 for the ESCI and ESCI-U other. Meanwhile, average variance explained ranged from 0.521–0.697 for the ESCI and ESCI-U self and 0.549–0.720 for the ESCI and ESCI-U other.

A number of studies have shown a statistically significant prediction or association with effectiveness and a variety of desired outcome measures of leaders, managers and professionals. Bajaj and Medury (2013) studied Indian software managers (*n* = 156). They showed that behavioral EI (i.e., aggregated others’ scores from the ESCI) predicted leadership effectiveness and transformational style using the Multifactor Leadership Questionnaire (MLQ). Pardasani (2016) showed that subordinates’ views of their leader’s ESCI predicted the leader’s psychological well-being, engagement and organizational virtuousness in 222 Indian dyads. Miller (2014) studied next generation leaders in family businesses. He showed that others’ views of the leaders predicted leadership effectiveness. Among professional leaders, Mahon et al. (2014) showed that behavioral EI among 231 knowledge workers in teams predicted their engagement. In institutions of higher education. Babu (2016) showed that for 100 community college presidents, behavioral EI predicted cognitive and emotional engagement and effectiveness as assessed with the Reputational Effectiveness Questionnaire.

Product innovation was strongly related to EI as seen by subordinates in 105 research and development executives from high technology companies (Kendall, 2016). In addition to exploratory and exploitative product innovation, and product innovation success, behavioral EI predicted competitive organizational performance and relative market share. The latter two effects were mediated by the quality of relationships at work in terms of degree of shared vision, compassion and relational energy. This suggests that individual capabilities like EI would be expected to be modifying (i.e., statistically speaking mediating or at least moderating) the predictive relationship to outcome variables.

Aliaga and Taylor (2012) found that effective Peruvian managers showed more behavior EI than their less effective counterparts. In the military, Koman and Wolff (2008) showed that 81 team leaders (i.e., outstanding commanding officers among flight crews and outstanding crew maintenance team leaders) distinguished themselves with behavioral EI. Young and Dulewicz (2009) showed similar patterns with 261 British Naval Officers with a different 360^°^ assessing behavioral EI competencies. In a study of executives in a major US bank, Hopkins and Bilimoria (2008) showed that among 105 executives, behavioral EI distinguished the more successful in terms of performance ratings. An interesting side note, they also showed that there were no significant differences in the behavioral EI shown among the male and female executives, although gender did moderate the effect of using the behavioral EI on success.

One criticism of the concept of EI is the redundancy of measures of EI with general mental ability (i.e., *GMA* or cognitive intelligence) and personality traits. Using a sample from the research engineering division a large, multinational automotive company, behavioral EI (i.e., scores on the ESCI as seen by peers in their project teams) accounted for a significantly high unique variance (Δ*R*^2^ = 0.31, *n* = 40) in effectiveness of the engineers. General Mental Ability (GMA) assessed with the Ravens Progressive Matrices (RPM) (Ravens, 1962) and personality traits assessed with the NEO-FFI (McCrae and Costa, 2010) did not (Boyatzis et al., 2017). In another study of financial sales executives, behavioral EI (i.e., as measured by peer and subordinate reports on the ESCI) also accounted for a significant unique variance in the measure of leadership effectiveness beyond the impact of *GMA* again assessed with the Ravens Progressive Matrices and Big Five personality traits assessed with the NEO-FFI (Δ*R*^2^ = 0.03, *n* = 60, Boyatzis et al., 2012).

Using a different measure of *GMA*, namely the Dental Admissions Test (DAT), no significant association was found with behavioral EI among graduate students in dentistry (Victoroff and Boyatzis, 2013). While the DAT score did predict grades in the first 2 years of dental school which are made up of only didactic courses, it was behavioral EI that predicted the grades in the third and fourth years which are all in the clinic.

In a Bayesian analysis of distributions, Boyatzis et al. (2015a) reported that distributions of GMA (i.e., as approximated through GMAT scores) showed that observations using the ESCI-U was clearly different than cognitive competencies. The data was from 641 MBAs at a top ranked MBA program with students from 23 countries in the sample. The CFA of the ESCI-U confirmed appropriateness of fit with RMSEA (RMSEA = 0.04 for professional sources and 0.05 for personal sources) and CFIs (0.99 for professional sources and 0.99 for personal courses) at desired levels. It supports the results from the studies reviewed above that showed behavioral EI is neither consistently related to measures of cognitive intelligence, nor a reflection of it.

## Distinctive Benefits of the Behavioral Level

### Self-Assessment Versus Other Reports (i.e., 360°)

The challenges of self-assessment plague the Stream 2 and 3 measures of EI, but not Streams 1 and 4. Podsakoff and Organ (1986), Tsui and Ohlott (1988), and Taylor (2014) reported the same observation that Hollander reported first in the 1950’s which is that leader’s self-assessment of their behavior and skills was inadequate. Later scholars wondered whether there was some contamination because of narcissistic delusion (i.e., people being legends in their own mind). Behavioral measures (i.e., whether codes for observing live, audiotaped or videotaped samples of behavior or 360° assessment) should be determined by inductive research. The validated codes become items in the 360°. Therefore, the resulting competencies are observable by others. This avoids the perceptual difficulties and possible biases of self-assessment. But it does raise the question as to whether self-assessment and other-assessments relate to similar outcomes.

In a first of its kind study of next generation leaders in family businesses, Steven Miller (2014) examined 100 next generation leaders from 100 family businesses. Among a variety of other variables, Miller (2014) collected 360° data from 350 other family and non-family business executives. EI was assessed by the ESCI (Boyatzis and Goleman, 2007) from these “others” as well as self-assessed by the next generation leader. Two dependent variables were used: (1) leadership effectiveness was assessed by others using the Leadership Effectiveness Scale (Denison et al., 1995); and (2) engagement was measured with the Utrecht Work Engagement Survey (UWES) (Schaufeli and Bakker, 2004). All scales used showed appropriate psychometrics in exploratory factor analyses, confirmatory factor analysis model fit, and appropriate indexes of convergent and divergent validity (Miller, 2014).

The results of this SEM showed a divergence of effect of the behavioral and self-assessed EI. Behavioral EI had a highly significant relationship (β = 0.64, *p* < 0.001, *n* = 100) with perceived leadership effectiveness and no relationship with engagement (Miller, 2014). In the same SEM, self-assessed EI from the ESCI showed a significant beta with engagement (β = 0.48, *p* < 0.001, *n* = 100) but showed no relationship to perceived leadership effectiveness. Furthermore, when he calculated a measure of self-awareness often used in the literature (the EI competencies self-assessed score minus the other-assessed aggregate score), it showed no relationship to perceived leadership effectiveness but a significant, negative relationship with engagement (β = -0.21, *p* < 0.01, *n* = 100).

Of course, a possible source of bias was from the collection of the behavioral EI and effectiveness measures from others, and the self-assessed EI and engagement from the leader, themselves. This would not have the same bias effect on the self-awareness variable.

Miller (2014) entered a hypothetical common latent factor in the SEMs, and compared the loadings with and without the common latent factor to test the possibility that common source bias affected the results (Podsakoff et al., 2003). No differences beyond the accepted indicator of.20 loadings were found. So common source bias was less likely of a factor in these results.

As a guide to future research, if the differences between the relationship of behavioral measurement of EI and self-assessment to outcome measures approximates these results in future studies, and based on earlier studies showing significant differences in accuracy, it would seem that behavioral EI measures would have a stronger and more consistent relationship to life and work outcomes than self-assessment measures.

### Quality of Relationships Matter

Emotional intelligence expresses itself through our relationships. Another way to say this would be that EI exists and is used in a context. The context of most relevance to how EI emerges is the relationships within which we are operating in a situation. We can expect that the quality of a relationship with someone trying to help us does make a difference, as Rogers (1961) claimed many years ago. Ellen Van Oosten found exactly this in her study of 85 top executives in a major bank (Van Oosten, 2013). She found that two clusters of behavioral EI predicted three different performance measures. A cluster she called Emotional Acumen (accurate self-assessment, empathy, emotional self-awareness, emotional self-control, teamwork, transparency, optimism) predicted the executive’s own degree of engagement and career satisfaction. Another cluster of EI competencies which she called Change Leadership (achievement orientation, change catalyst, initiative, inspirational leadership, self-confidence) predicted the boss’s performance ratings of that executive but negatively predicted the executive’s own career satisfaction.

Each executive in this study was provided with a professional coach. Van Oosten (2013) found that if the relationship the executive had to their coach was seen as having a high degree of shared vision, compassion and positive mood, the effects (i.e., the beta coefficients) predicting leadership effectiveness were increased. The quality of the relationship with the coach moderated the impact of behavioral EI on effectiveness measures. This same characteristic of the quality of relationships within the family business climate had a similar moderating effect on leadership effectiveness and engagement for next generation family business leaders (Miller, 2014).

In a study of teams of professional researchers in a manufacturing and a consulting company, Mahon et al. (2014) showed that degree of perceived shared vision in their relationships in the research teams also moderated the impact of behavioral EI on engagement. The study of behavioral EI of community college presidents cited earlier found the same pattern. Babu (2016) showed that perceived degree of shared vision and compassion partially mediated the President’s behavioral EI as it affected their perceived effectiveness from direct reports and their own engagement.

### Utility of the Behavior Level

Since most training, education and coaching predominantly attempt to help people change the way they act, the behavioral level of EI and measuring it can be directly useful. It provides feedback and guidance about how to act and how to change your actions to be more effective, innovative or satisfied and engaged with others (Cherniss, 2010). Changing how a person’s acts with specific behavior is in the realm of feasible development without resorting to multi-year efforts (Kanfer and Goldstein, 1991). Although evidence suggests some personality traits are malleable with specific interventions (Roberts et al., 2017), changing one’s traits seem to require a major effort. A person does seem to be able to change their levels on personality traits, like the Big Five, but these changes often take years, aging and/or a major change of setting in life and socialization into those new settings. Most psychologists contend that one can more easily change behavior (Kanfer and Goldstein, 1991), as has been shown in cognitive behavioral therapy versus other approaches to psychotherapeutic change (Barlow, 1988; Hubble et al., 1999).

Behavioral level measures of EI are used in many companies and government agencies in multiple countries as a key element in training programs (website for the Consortium for Research on Emotional Intelligence in Organizations, CREIO). They are often used by executive coaches throughout the world. The CREIO reported finding 15 model programs in an exhaustive review of published studies of change in EI anywhere in the world between 1950 and 1996 (Cherniss and Adler, 2000). In every study, the desired change was in a person’s specific behavior of EI competencies. Of those reported, only five had continued to be in use in 1996. Two of those were the programs for EI competency development at Alverno College (Mentkowski and Associates, 2000) and CWRU (Boyatzis et al., 2002).

Because of its focus on behavior, this approach to EI is used in many colleges and universities in a variety of countries as part of their undergraduate and graduate courses on leadership in management, medicine, law, dentistry, engineering, arts and sciences, social work, and other programs (Boyatzis and Cavanagh, 2018). Management schools use behavioral measures of EI in executive education or continuing education programs for the same reason. Statistically significant improvement of students’ behavior has been shown in many articles in a variety of these fields since 1996 (Boyatzis et al., 2002) using behavioral EI.

## A Multi-Level Theory of EI

### Stream 4 as Behavioral EI

In search of a model of EI, scholars have been writing about EI in terms of a model with Streams 1, 2, and 3 as proposed by Ashkanasy and Daus (2005) (O’Boyle et al., 2011; Joseph et al., 2015; Miao et al., 2016b) and now about an additional Stream 4 (Amdurer et al., 2014). The Ashkanasy and Daus (2005) framework is really a classification of EI measures. According to Ashkanasy and Daus (2005), Stream 1 EI measures a person’s intellectual ability with emotional information using a four branch model as assessed in the MSCEIT (Salovey and Mayer, 1990; Mayer and Salovey, 1997). It attempts to be a direct ability measure of how a person is processing emotional information. In their typology, Stream 2 measures are self-assessment measures of the model based on the MSCEIT, such as the SREIS (Schutte et al., 1998) and WLEIS (Wong and Law, 2002; Law et al., 2004).

In the Ashkanasy and Daus (2005) typology, Stream 3 were all other measures, including traits, like the TEIQUE (Petrides and Furnham, 2000, 2001) and the EQ-I (Bar-On, 1997). This classification confused assessment of traits, self-schema and behavior as parts of one approach. In their original model, they included what this author and others are now claiming constitute a Stream 4. In other words, the Ashkanasy and Daus (2005) Stream 3 is now proposed to be split into a new Stream 3 of trait and other self report measures based on models of EI other than the MSCEIT, and into Stream 4, which would be the behavioral measures based on a variety of EI models.

Stream 4 EI measures are those that assess a person’s behavior (Amdurer et al., 2014). The key source of the information is from others or direct observation and coding. In the proposed Stream 4 measures, EI is operationalized as informants’ or direct observations by others of a person’s behavior through the ESCI, other 360 measures (i.e., the 360° version of the WLEIS or EQ-i, or Dulewicz et al., 2003) or coding from audio tapes of critical incidents (Spencer and Spencer, 1993; Ryan et al., 2009) or videotapes of simulations (Boyatzis, 2017). In some research the same test may be used to study Stream 3 and proposed Stream 4 EI by using the self-assessment version of a 360 and the informants’ responses, respectively. In the case of 360’s, the distinction of Stream 3 and 4 is more focused on the source of the data than the specific items or structure of the measure.

The need for this distinction of Stream 3 and 4 measures is apparent in a variety of meta-analyses showing that Stream 1 measures (i.e., the MSCEIT) do show a significant correlation with cognitive intelligence or GMA. As discussed earlier, results from behavioral measures do not show any consistent significant relationships to personality traits in terms of the Big Five or GMA (Boyatzis, 2017). Meta-analyses have shown consistently that EI measures do predict job performance (Joseph and Newman, 2010; O’Boyle et al., 2011; Joseph et al., 2015), job satisfaction and subordinate job satisfaction (Miao et al., 2016a,b), and organizational citizenship behavior and negatively with counter productive work behavior (Miao et al., 2017). Often Stream 2 and 3 measures showed stronger unique variance or relative importance than Stream 1 measures (O’Boyle et al., 2011; Miao et al., 2016a,b, 2017). But Stream 3 measures were seen to conflate EI with a mixture of concepts including personality traits and intelligence (Joseph and Newman, 2010; Joseph et al., 2015). Joseph et al. (2015) cited studies showing a strong link between self-report measures of EI and conscientiousness and extroversion. One measure of EI, the SREIT, showed high correlation with Openness (*r* = 0.54), but the absolute correlations with other Big Five personality traits were lower (*r* = 0.21–0.28) (Schutte et al., 1998). But in another study, the SREIT’s correlations with the Big Five personality traits ranged from 0.18 (Agreeableness) to 0.51 (Extraversion) (Saklofske et al., 2003). Bar-On (1997, p. 16) mentioned that the EQ-i overlaps with personality “probably no more than 15% based on eight studies in which more than 1,700 individuals participated.” However, one study showed that the Big Five predicts EQ-i scores with a multiple correlation of 0.79. This suggests that the Big Five accounted for the majority of variance in the EQ-i (Grubb and McDaniel, 2007).

The need to split the earlier category of Stream 3 into Stream 3 and 4 measures became clearer as deeper examination of the link of Stream 3 to job performance was claimed to be more a function of these other variables than EI (Joseph et al., 2015). This reflected analogous observations from Webb et al. (2013) showing that measures of Big Five personality traits and GMA accounted for 2/3rds of the variance of the EQ-i but only 14% of the variance in the MSCEIT. In addition, an earlier article critical of the mixed models measures claimed that there were likely to be two different constructs among these measures (Van Rooy et al., 2005).

### A Multi Level Theory of EI Is a Domain Specific Theory of Personality

One of the main ideas in this paper is that a multi-level theory of EI is a multilevel theory of personality applied to the specific domain of EI. According to Merriam Webster Dictionary (2018) (January 1), personality is, “the complex of characteristics that distinguishes an individual or a nation or group: the totality of an individual’s behavioral and emotional characteristics.” We can further support this complex notion of personality by the definition offered by the American Psychological Association (American Psychological Association [APA], 2018) itself, “personality refers to individuals differences in characteristics patterns of thinking, feeling and behaving.” The explanation from APA goes on to claim that the “study of personality is of its parts and how they fit together as a whole.” Allport (1937) defined personality as, “the dynamic organization within the individual of those psychophysical systems that determine his unique adjustments to his environment.”(p.48). In depicting the major components of personality, McClelland (1951, p. 595) he described personality as having a need or motive underlying system, a schema which also includes a self-schema, a trait system by which he means an “activated habit family hierarchy” and predicted concrete acts (i.e., behavior).

All of these definitions portray personality as a complex of characteristics that interact and result in specific behavior. McClelland’s view provided a multi-level image that began with deeply unconscious motives and needs, rising through one’s conscious and semi-consciousness into a schema (and self-schema), emerging as behavioral habits which he called traits and then appearing in concrete actions or behavior. Later, he conceptualized “competencies” as sets of functionally related skills (McClelland, 1973).

Boyatzis (1982) expanded on McClelland’s personality model to suggest that neural and hormonal dispositions, along with motives and traits, constituted the deepest level of one’s personality which was primarily unconscious. He further postulated that the self-schema emerged as a result of these unconscious characteristics interacting with social groups and cultures (social identity groups and normative cultures, both local and general) to generate a sense of self which incorporated the values to which one aspired, both core and contingent values. This domain of personality was conscious, subconscious and somewhat unconscious, depending on the degree of the person’s self-reflection and introspection. At the conscious and observable level (i.e., others around a person can see it), the person’s motives, traits, physiological dispositions, self-image and social identity emerged as a set of actions (Cherniss, 2010; Cherniss and Boyatzis, 2013). When these actions were a pattern of behavior and generalized across settings and stimuli, they formed competencies (Boyatzis, 1982). These were a set of related skills with an underlying (i.e., often unconscious) intent. By expanding the model of EI to include a behavioral level, an additional contribution of this paper is to offer a holistic or comprehensive model of EI by focusing on the heretofore under addressed behavioral level in the EI literature.

**FIGURE 1:**
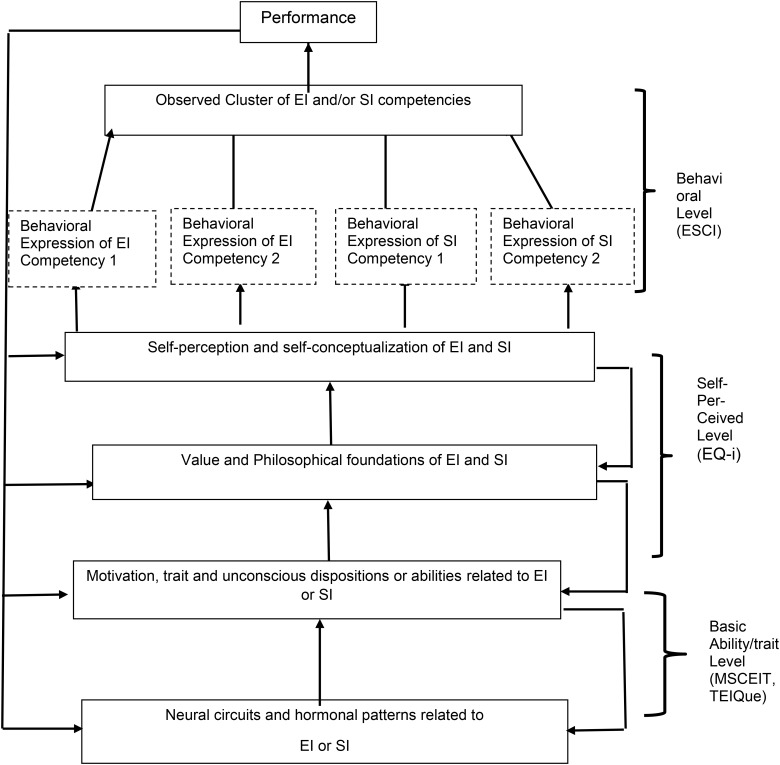
Emotional intelligence (EI) as a multi-level theory [from Cherniss and Boyatzis (2013) which was adapted from Boyatzis (1982, 2009)].

The application of this multi-level theory of personality to the specific domain of EI is shown in **Figure 1**. It shows how the various concepts or theories of EI as well as various measures can be portrayed as components or levels of the individual’s EI.

### Another Multi-level Theory Related to the Cognitive Intelligence Domain

Beyond the concepts of crystalized and fluid intelligence and working memory, *g* and GMA, even within the domain of a psychological concept like cognitive intelligence, multi-level theories have emerged with behavioral levels proposed. One approach was Sternberg’s (1985, 2011) “triarchic theory” by purporting that internally, a person has “meta-components, and knowledge-acquisition components” (Sternberg, 1985, p. 59). It includes a wide range of analytic processes in thinking about life. Sternberg (1985, 2011) proposed that the application of these intelligences to everyday life constituted a “practical intelligence” (Sternberg and Hedlund, 2002), which he later subsumed as part of a larger concept of “successful intelligence” (Sternberg, 1999) and within that a social intelligence (Sternberg, 2011).

His theory contended that analytic processes were only one part of a person’s ability to “adapt to the environment and learn from experience” (Sternberg and Detterman, 1986). Successful intelligence was the inclusive concept that incorporated goals for one’s life and work. It included crystalized and fluid intelligence and added elements from what was later to be called EI. Meanwhile, practical intelligence was the use of tacit knowledge gained from one’s experiences. Practical intelligence, measured through tacit knowledge, was directly related to performance of leaders in management simulations (Sternberg, 2011), and leadership effectiveness while controlling for *g* (Sternberg and Hedlund, 2002; Hedlund et al., 2003). A major contributions of this approach was to bring a behavioral level to the concept of intelligence. People could now talk about, theorize, and study how individuals applied their internal, cognitive capability and how it looked to observers.

Another multi-level approach further challenged the role of cognitive intelligence was Grossman et al. (2013) concept of wise reasoning. It was defined as pragmatic analysis in social settings, especially within emotional and conflict events, predicted well-being, career and life satisfaction, and longevity. In their studies, measures of traditionally defined cognitive intelligence were negatively related to wise reasoning and well-being. They went further to claim, as shown in prior research, that cognitive abilities such as crystallized intelligence, processing speed, and working memory showed no systematic, positive relationship to well-being. The entrance of reasoning into the behavioral realm provided further insight when Brienza and Grossmann (2017) showed that wise reasoning was inversely related to current social class, suggesting an important possible moderator or control variable for future studies.

## Measurement Challenges of the Behavioral Level

The most persistent challenge of research at the behavioral level is the time intensive operant methods of collecting and processing qualitative information. Operant methods ask the person to react to and act on relatively ambiguous stimulus, like the Thematic Apperception Test or the Roschach Ink Blots. Structured and semi-structured interviews allow the person responding a wide range of options in what and how they say and act. This is in contrast to respondent methods, like multiple choice or Likert scaled surveys in which the person is forced to respond among a fixed set of choices. As mentioned earlier, both conducting interviews and/or convening simulations, is just the beginning. Then follows hours and hours of coding of audio, video tapes and transcripts. Maintaining reliability of both the interviewers and coders is a methodological problem.

Although 360° assessments have been used for 6 decades, their use in research is much more recent. In the past, consultants (and some professors) would develop items for 360° and then use the tests without engaging in the appropriate psychometric testing. Any assessment should have replication of exploratory factor analyses, confirmatory factor analyses, computation of convergent and discriminant validity, as well as a series of validation studies against a variety of performance measures and dependent variables. In addition to published papers, all of the dissertations reviewed in this article using the ESCI or ESCI-U have established all of these properties of sound psychometric rigor.

In using 360° measures in research, authors will aggregate multiple others’ (or informants) observations into a single score. A controversial issue that plagues 360 measures involves justifying the aggregation of scores into an “others” view. Traditionally, an aggregation required documenting intraclass correlations (ICCs). But intraclass correlations assume you should only aggregate scores of responses that are highly correlated. Often, when using 360, the intent is to generate a composite of how others in the person’s environment see their behavior. One of the primary goals of using 360 and collecting observations from informants that have different perspectives on the focal person (i.e., subordinates, peers, bosses and customers, and possibly even personal relationships like spouses/partners and friends) is to collect a variety of perspectives. These different views may generate more valid descriptions of a person’s overall comportment because they are not highly correlated. One study showed that we should use non-correlation based indicators like factorial, scalar and measurement invariance methods (Batista-Foguet et al., 2014) which reiterated some of the same arguments made by de Vijver and Tanzer (2004) about establishing desired invariance across cultures. Their contention is that these assessments of invariance are far more comprehensive and less susceptible to distortion of extreme scores, which with correlation-based methods may result in inflation of effects.

Another challenge was uncovered regarding response set differences between people from European countries and others. In Europe, ten-point scales (i.e., decimal) are used for everything, from market research to grading at universities. In a carefully designed study with random assignment and alternating sequence of forms, it was found that for Europeans, an eleven point scale (i.e., one point for “don’t know”) created less invalidity. This stands in contrast to other parts of the world where five or seven-point scales appear more reliable (Batista-Foguet et al., 2009).

Other challenges include whether different sources are equally reliable sources of observation of a person’s behavior. The contention of many 360° users is that a more comprehensive set of sources provides a more thorough review of the person’s variety of behavior. Understanding how a person acts at work may help in their development, but understanding if the person uses the competency behavior at home or in leisure settings provides a more thorough review of the person’s range of action. It also suggests different tactics in helping the person change their behavior in either work or personal settings. Boyatzis et al. (2015a) showed that personal sources, like friends and spouses view a quite different range of a person’s behavior than professional sources or people at work. There was a dramatic gender difference in these effects in particular among professional sources in which observations of males behavior was internally more consistent and did not show distorting distributions in a Bayesian analysis.

### Clusters of Behavioral EI

Although the issue of scale construction and even greater aggregations into clusters of EI plagues each of the measures, with behavioral EI it becomes more than a statistical exercise. Boyatzis (1982) claimed that clusters can be developed as theoretical or empirical. For example, in behavioral EI, one cluster was thought to be self-awareness, another self-management, another social awareness and another relationship management. Coded competencies from audiotapes of critical incidents at work or videotapes of group simulations (Boyatzis, 1982) or 360° assessment (Boyatzis and Sala, 2004) revealed that the empirical clusters do not neatly follow the theoretical predictions. Boyatzis and Sala (2004) They reported two dominant clusters with results from an earlier test, the ECI-2 of: (1) emotional self-awareness, accurate self-assessment, transparency, empathy, developing others and teamwork; and (2) achievement orientation, inspirational leadership, change catalyst and optimism. These clusters had about half of the competencies loading into clusters empirically that were different from those predicted theoretically.

While a critique could be that it suggests the theory is wrong, another possibility is that the specifics of an industry or even a strong company climate might change the way people use the competencies. The EI competency of emotional self-control might be theoretically interpreted as a personal, self-management capability. But in banks where a financially oriented set of measures of performance is a preoccupation, it might be a set of behavior demonstrated along with empathy, teamwork but not inspirational leadership (Van Oosten, 2013).

Among the bank executives, Van Oosten (2013) showed that such a cluster predicted engagement and career satisfaction. She had two dominant clusters also from the ECI-2 from the bank executives of: (1) emotional self-awareness, accurate self-assessment, emotional self-control, transparency, teamwork and optimism; and (2) achievement orientation, change catalyst, inspirational leadership, and self-confidence. These clusters overlapped with the theoretical clusters about 2/3rds of the time. In a different setting and industry, like community colleges, presidents might use emotional self-control along with empathy, teamwork and inspirational leadership due to the nature of higher education being a professional service where their outcomes are not measured in financial returns but on whether a graduate feels more capable and is able to get a desired job. As stated earlier, but with regard to clusters, among community college presidents (Babu, 2016), this cluster had a direct effect on engagement, whereas the other cluster of behavioral EI affected engagement but was fully mediated by the quality of the relationship mentioned above.

## Conclusion

A comprehensive and holistic view of EI should include the multiple levels of EI. Behavioral EI is an approach that could complement other levels and measures and potentially offer stronger and unique variance of predicting job and life outcomes, performance, engagement, citizenship and innovation. First, the behavioral level must be treated as a separate form of EI, with its own types of measures. Second, behavioral measures along with measures of other levels of EI should be tested against job and life outcome measures, controlling for GMA and personality. Third, the same study designs should be replicated with various moderator or mediator variables. Although quality of relationships was examined in this paper, there are numerous other potential variables of interest, such as self-efficacy, emotion perception abilities, wise reasoning and practical intelligence, and so forth. Prospective mediators could include other forms of information processing as well as perceptual variables.

Fourth, to use a behavioral level information, use of 360° assessments is most likely in quantitative studies. As we suggested, a few methodological challenges must be investigated further. We raised initial studies about response set and sources. Regardless, the basis for aggregation should move away from traditional correlation based methods like ICCs and toward the three sources of invariance.

All of this suggests a higher sensitivity to domain specific models and using a variety of appropriate measures. Just as the domain of cognitive intelligence has evolved as a complex array over the last 100 years, we can expect emotional and social intelligence to continue along a comparable path. Few psychology Departments in research oriented Universities exist today without numerous neuroscientists. We have come a long way from stimulus-response-reinforcement explanations for human behavior. The increased complexity should not obscure or be used to avoid good science. The increasingly complex concepts must be assessed separately and together to create more holistic theories. Given the demands of specialization in each of our fields and specialties, this suggests the need for more research teams composed of scientists with different disciplinary backgrounds.

## Author Contributions

The author confirms being the sole contributor of this work and approved it for publication.

## Conflict of Interest Statement

The author has a proprietary interest in one of the several behavioral measures discussed in this paper. All measures including this one are available for free use by faculty or student researchers from the publisher.
